# Inverted Papilloma of the Middle Ear and Mastoid Cavity: A Case Report, Literature Review, And Surveillance Proposal

**DOI:** 10.51894/001c.7406

**Published:** 2019-03-04

**Authors:** Christopher M. Metz, Robert T. Standring, Seilesh C. Babu, Christian E. Keller

**Affiliations:** 1 Osteopathic Division; St John Providence Health System, Madison Heights, Michigan; St. John Providence Otolaryngology Division, Novi, MI; 2 St. John Providence Otolaryngology Division, Novi, MI; Ear Nose and Throat Consultants, Southfield MI; 3 Department of Otolaryngology–Head and Neck Surgery; Neurotology Division; Department of Surgery St. John Providence Otolaryngology Division, Novi, MI; Michigan Ear Institute, Farmington Hills, MI; Wayne State University, Detroit, MI; St John Providence Health System, Novi, Michigan; Oakland University William Beaumont School of Medicine, Rochester, Michigan; 4 Department of Pathology Henry Ford Hospital, Detroit, MI

**Keywords:** pediatric skull base surgery, pediatric otology, inverting papilloma, pediatric middle ear masses

## Abstract

**INTRODUCTION TO THE TOPIC:**

Inverted papilloma is a rare condition of the middle ear. In this paper, the authors present a case report of a patient at a Midwestern health system with inverted papilloma. To supplement the case report, a literature review was also performed to identify clinical trends predisposing such cases to recurrence, malignant transformation, and response to radiation. In addition, the authors also propose a surveillance algorithm derived from this case and previously published surveillance strategies.

**CASE REPORT:**

The authors present a rare case of inverted papilloma of the middle ear. To the authors’ knowledge, this is the youngest case presentation (mid-teenage years) of this condition to have been reported in the literature. The patient underwent surgical excision, had recurrence, and has been disease free since revision surgery.

**SUMMARY OF THE EVIDENCE:**

Our literature review identified 25 cases previously published with ours being the 26th. An inadequate number of cases exist to abstract statically relevant clinical trends in presentation and tumor behavior. Additionally, no tumor characteristics have been identified that predispose tumors to future malignant transformation. No assessments can be made regarding the benefits of radiation therapy. Most cases to date have been surveyed with a combination of CT, MRI, and clinical follow-up.

**CONCLUSIONS:**

Inverted papillomas of the middle ear space are rare. Although this case report adds to the literature, additional cases are needed to draw statistically relevant clinical characteristics and responses to medical and surgical therapy.

## INTRODUCTION

Inverted papillomas are benign tumors typically found in the nasal cavity. These locally aggressive tumors have a potential for malignant transformation. Presentations of inverted papillomas in the middle ear space are rare, with the previous literature reporting as few as 23 cases total.^1^ In this paper, the authors will report a case of a recurrent inverted papilloma of the middle ear space and present a comprehensive literature review of previously reported cases of this type of inverted papilloma. Finally, a surveillance algorithm-based protocol will be proposed for monitoring of recurrence.

## CLINICAL CASE

A female in her mid-teens presented with a chief complaint of hearing loss. Initial otoscopic examination revealed a bulging tympanic membrane with an inflamed mass occupying the middle ear space. Nasopharyngoscopy (i.e., an endoscopic exam of the nasal cavity) did not reveal any sinonasal masses or lesions. A hearing test demonstrated a unilateral, profound hearing loss. (Figure 1)

**Figure:**
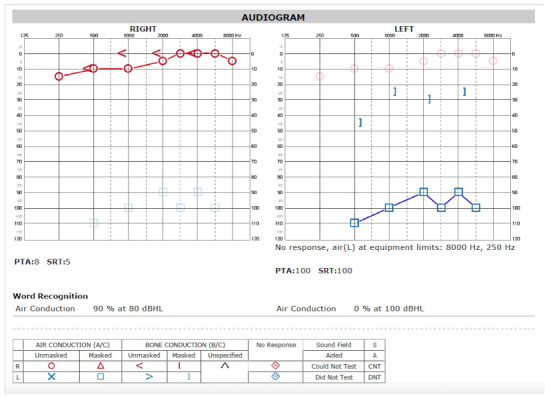
Figure 1 Audiogram Obtained at Presentation. Graphs Show Unilateral, Left, Mixed Profound Hearing Loss

A CT scan of the temporal bones showed nonspecific, complete opacification of the middle ear and mastoid on the affected side. (Figure 2) An additional MRI scan was obtained showing an enhanced soft tissue mass centered within the left middle ear cavity. No intracranial involvement was noted.

**Figure:**
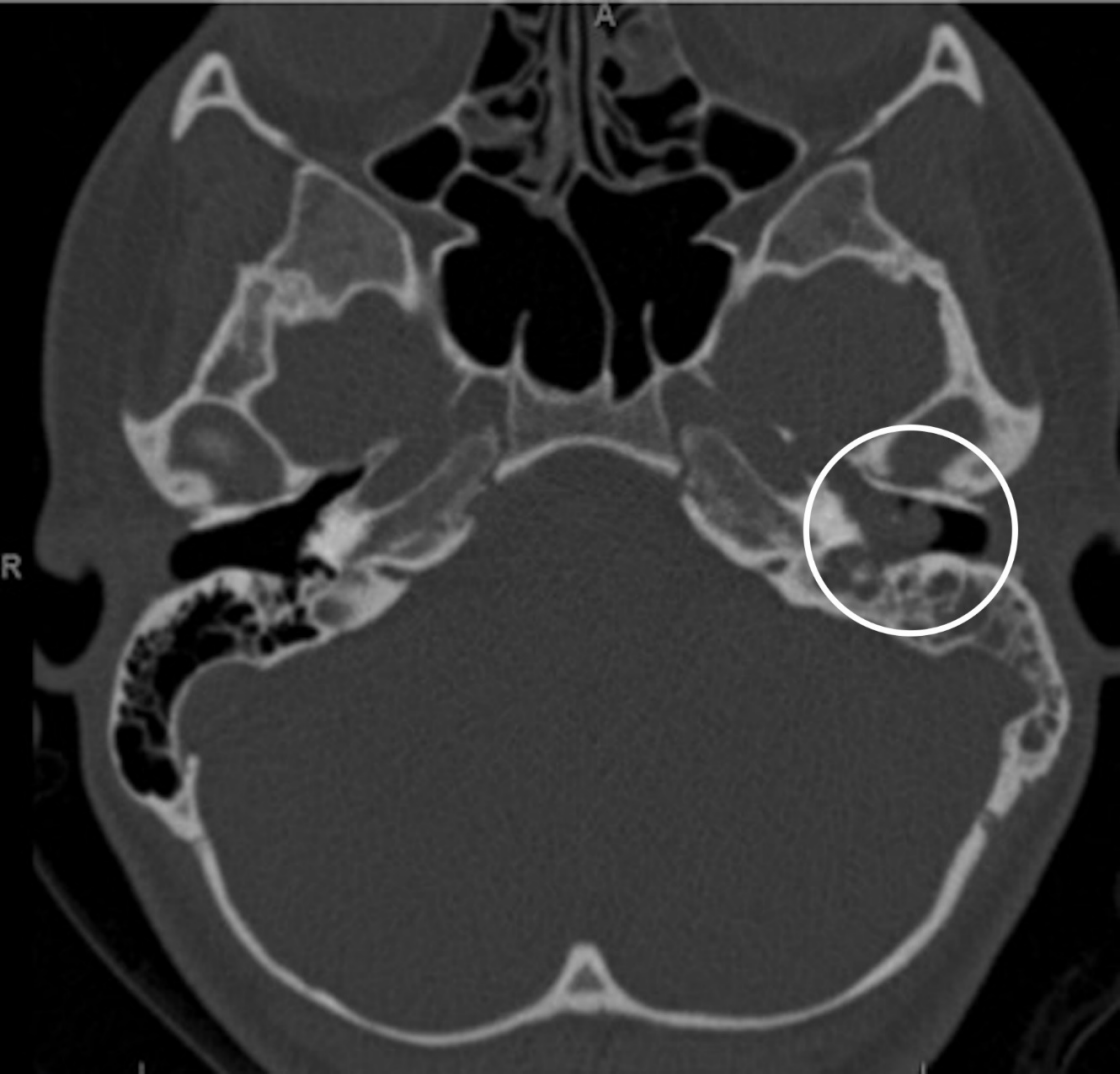
Figure 2 Axial-cut CT Showing Left Middle Ear Mass

As seen in Figure 3, there was a proliferation of thickened transitional-type epithelium with an inverted growth pattern, forming well-circumscribed lobules and glands that emptied onto the luminal surface. No evidence of infiltrative growth or necrosis was seen. On higher power (inset) the neoplastic cells had features of columnar and stratified squamous cells lacking significant mitotic activity or nuclear pleomorphism. Intraepithelial polymorphonuclear neutrophilic granulocytes were noted, which focally form microabscesses.

**Figure:**
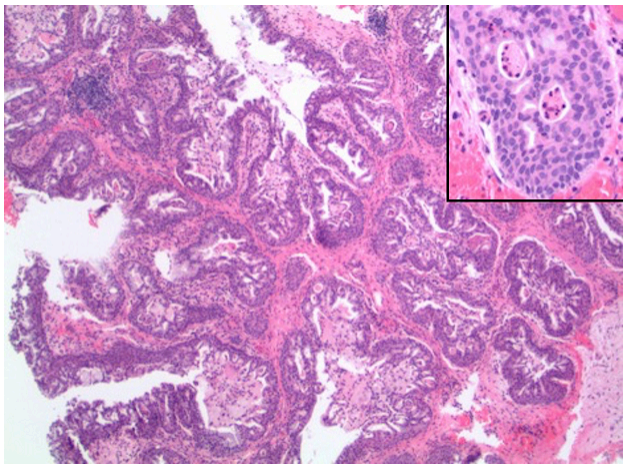
Figure 3 MRI of Left Middle Ear Cavity: Proliferation of Thickened Transitional-type Epithelium with an Inverted Growth Pattern, Forming Well-circumscribed Lobules and Glands that Empty Onto the Luminal Surface

The patient was brought to the operating room for a middle ear exploration and biopsy. A red, flesh-like mass was noted to be completely occupying the middle ear space. The mass appeared to be highly vascularized with finger-like projections extending radially. At the time of this exploration, the ossicular chain (i.e. the hearing bones) was completely encompassed in tumor and we were unable to ascertain proper anatomy and movement.

The Eustachian tube, (i.e., the narrow passage that leads from the pharynx to the cavity of the middle ear and permits the equalization of pressure on each side of the eardrum), could not be adequately viewed. A specimen of the mass was taken for pathologic review. Final pathology was interpreted as an inverted papilloma with no evidence of dysplasia (i.e. abnormal cell types suggestive of a malignant process).

After further discussion and planning by the authors, the patient underwent a surgical middle ear exploration with removal of the lesion and hopeful exteriorization. During the case, a 1 cm area of erosion of the bony eustachian tube was noted and subsequently, packed. The tumor was completely excised with the exception of two areas. A microscopic tumor was left as it was overlying facial nerve. Also, several microscopic tumor fragments were left affixed to the stapes around the oval window. Postoperatively, the patient did very well with no facial nerve weakness, although her hearing remained poor on the diseased side.

After 18 months of follow-up observation, she developed further hearing loss and ear drainage in the affected ear. A subsequent MRI demonstrated a tumor enhancement in the left mastoid region and left middle ear cavity. In addition, no enhancing lesions in either internal auditory canal were observed. No other lesions were noted in either the nasopharynx or neck.

The patient then underwent a revision modified radical mastoidectomy. Granulation tissue was noted in the mastoid cavity. There were some areas of inflammation and pockets of purulent (i.e., pus-filled) material, which were removed. Biopsies were obtained from the remnant tissue around the stapes and the facial nerve and were confirmed to be inverted papilloma. Small areas of remnant tumor along the facial nerve were left alone. She had normal postoperative facial nerve functioning. Her ear canal has since become fibrosed, scarred, and created an overclosed ear canal.

The patient has been asymptomatic since revision surgery with stable hearing loss. A pair of postoperative MRI films performed at one and six months after the second surgery showed a clear mastoid cavity with no evidence of recurrence.

## SUMMARY OF THE EVIDENCE

The authors conducted a literature review to identify a total of 25 previously published cases of inverted papilloma of the middle ear and mastoid cavity, ours being the 26^th^. Publication dates ranged from 1987-2016.^2,3^ (Table 1) Twelve (46.2%) of the 26 cases had a history of sinonasal papilloma. The average age at presentation was 51.7 years. Notably, our case presented in this paper has the youngest age at presentation (mid-teens). The other earliest published age for a patient with this condition had been in their late-teens.^4,5^ Variations of this condition have existed in both presentation and tumor behavior.

**Table:** Table 1 Summary of Previously Discussed Cases

**Author**	**Presenting Symptoms**	**Age at Diagnosis**	**Sex**	**Radiation**	**History of Nasal Papilloma?**	**Histology**	**Surgery**	**Recurrance**	**Monitored With**
**Schaefer**	**Hearing loss, otorrhea**	**46**	**Male**	**No**	**No**	**Schneiderian type papilloma, no evidence of malignancy**	**Radiocal mastoidectomy, repair of tegmen dehiscence**	**None reported**	**Not stated**
**Rubin**	**Hearing loss, otorrhea**	**73**	**Male**	**No**	**No**	**Papilloma, no evidence of malignancy**	**Open tympanoplasty**	**None reported**	**MRI/CT**
**Nath**	**Hearing loss, otorrhea**	**60**	**Male**	**Yes**	**No**	**Inverted papilloma with marked dysplasia**	**Radical mastoidectomy**	**Yes, 11 month post treatment**	**MRI**
**Stone**	**Otalgia, otorrhea**	**55**	**Male**	**Yes, after recurrence**	**Yes**	**Epithelial papilloma with focal atypia**	**Modified radical mastoidectomy**	**Yes, none after radical mastoidectomy and radiation**	**CT**
**Kaddour**	**Otalgia, otorrhea**	**87**	**Female**	**No**	**Yes**	**Transitional cell papilloma**	**None, patient poor surgical candidate**	**Not ressected**	**Clinically with occasional EAC debulking**
**Roberts**	**Hearing loss, otalgia**	**19**	**Female**	**No**	**No**	**Atypical inverted nests of epithelium**	**Tympanomastoidectomy with a facial recess approach**	**None**	**Serieal middle ear exploration**
**Seshul**	**Hearing loss, unilateral serous otits media s/p ressection nasally**	**31**	**Female**	**Yes**	**Yes**	**Inverted papilloma**	**Radical mastoidectomy**	**Yes, malignant transformation, multiple recurrances in ear and nasal cavity**	**Clinically/MRI/CT**
**Wenig**	**Conductive hearing loss, otalgia**	**31**	**Female**	**No**	**Unknown**	**Epidennoid papilloma with features of both inverted and cylindrical cell papilloma**	**Myringotomy with sim- ple surgical excision; radical mastoidectomy**	**Multiple**	**CT**
** **	**Otorrhea; polypoid mass protruding from middle ear**	**56**	**Female**	**No**	**Unknown**	**Epidermoid papilloma with exophytic and endophytic growth**	**Tympanomastoidectomy, ultimately necessitating radical mastoidectomy**	**Multiple**	**CT**
** **	**Chronic otorrhea**	**19**	**Female**	**No**	**Unknown**	**Epidermoid papilloma with features of cylindrical cell papilloma**	**Tympanomastoidectomy**	**None**	**CT**
** **	**Hearing loss, otaglia**	**57**	**Female**	**No**	**Unknown**	**Epidermoid papilloma with features of cylindrical cell papilloma**	**Myringotomy with simple surgical excision; treated by myringotomy and simple excision but ultimately necessitating radical mastoidectomy**	**Multiple**	**CT**
**Jones**	**Hearing loss, complete facial nerve paralysis**	**35**	**Female**	**No**	**Yes**	**Inverted papilloma, an extension of sinonasal disease**	**Fisch type C temporal bone ressection**	**No**	**Clinically**
**Chhetri**	**Aural fullness, hearing loss**	**26**	**Male**	**No**	**No**	**Epidermoid papilloma with features of cylindrical cell papilloma**	**Tympanomastoidectomy facial recesss approach**	**Yes**	**Not stated**
**Pou**	**Hearing loss, otorrhea**	**81**	**Male**	**Refused by patient**	**Yes**	**Carcinoma within the inverting papilloma**	**subtotal temporal bone resection**	**Yes**	**MRI +CT**
** **	**Hearing loss, otorrhea**	**54**	**Male**	**Yes**	**Yes**	**Inverting papilloma with squamouscell carcinoma**	**right-side subtotal temporal bone resection, sparing the oticcapsule and facial nerve**	**No**	**MRI + CT**
**de Filippis**	**Aural fullness, hearing loss**	**58**	**Male**	**No**	**No**	**Papillary neoplasia**	**Tympanomastoidectomy**	**No**	**MRI**
**Mazlina**	**Otorrhea**	**54**	**Male**	**Yes**	**Yes**	**Inverted papilloma with an area of malignant transformation**	**Patient refused**	**Not stated**	**Not stated**
**Ali**	**Hearing loss, otorrhea, tinnitus**	**42**	**Female**	**No**	**No**	**Exophytic papillomatous neoplasm composed of non-keratinized squamous mucosa with central fibrous core consistent with Schneiderian papillomatosis**	**Tympanomastoidectomy**	**No**	**CT**
**Acevedo-Henao**	**found on CT hx of sinonasal disease**	**63**	**Male**	**Yes**	**Yes**	**Inverted papilloma**	**Right subtotal petrectomy**	**Yes**	**MRI/CT**
**Inoue**	**Aural fullness**	**53**	**Female**	**No**	**No**	**Squamous papilloma without cell atypia.**	**Type I tympanoplasty and complete mastoidectomy.**	**Not stated**	**Not stated**
**Zhou**	**Otorrhea, diplopia**	**52**	**Male**	**Yes**	**No**	**High grade squamous intra- epithelial neoplasia**	**Canal wall down mastoidectomy, Fisch Type A temporal bone ressection. Temporalis muscle flap**	**No**	**Not stated**
**Shen**	**Aural fullness, hearing loss**	**56**	**Male**	**Yes**	**Yes**	**Inverted papilloma**	**Radical tympanomastoidectomy**	**No**	**CT**
**Kainuma**	**Hearing loss, otaglia**	**65**	**Male**	**Yes**	**Yes**	**Inverted papilloma with moderate atypia**	**Radical tympanomastoidectomy**	**Yes**	**Not stated**
**Mitchell**	**Middle ear mass**	**69**	**Female**	**No**	**Yes**	**Inverting Schneiderian papilloma with areas of squamous dysplasia and carcinoma in situ**	**Anterior skull base ressection, temporal bone ressection**	**No**	**MRI**
**Dingle**	**Aural fullness, hearing loss (bilateral)**	**52**	**Male**	**Yes**	**Yes**	**Invasive carcinoma with evidence of Schneiderian papilloma**	**Bilateral canal wall up tympanomastoidectomy**	**No**	**MRI**

Hearing loss appears to be the most common presenting symptom. Interestingly, there appears to be no correlation between severity of presenting symptoms and chance of recurrence. In 2012, Jones et al. described a case that presented with complete facial nerve paralysis, although no recurrence after resection was reported.^6^ Conversely, several cases of hearing loss as a presenting symptom have reported multiple recurrences despite surgical and medical management.^4^

Additional discrepancies exists in the literature regarding whether or not radiation therapy can decrease the probability of disease recurrence. Although radiation therapy has been shown to be an effective means of local control in some instances of sinonasal inverted papillomas,^7^ little evidence exists with regards to its role in treatment of middle ear papillomas. However, there were also multiple cases that presented recurrence despite aggressive radiotherapy.^8–10^ In 2002, Pou et al. describes a case in which post-operative radiation appears to have prevented known recurrence.^11^

Conflicts also exist regarding whether or not a history of sinonasal papilloma predisposes patients to more aggressive malignant forms of ear papillomas. Several previous reports have described patients with a history of nasal papilloma with malignancy as the original otologic histology.^5,8,11,12^ Alternatively, multiple cases have been presented with patients who have a strong history of sinonasal papillomas who never demonstrated any malignant transformation of otologic tumors.^6,10,13^

In this presented case of a patient in her mid-teens, many factors may have been relevant to her surgical outcome. During her first tympanomastoidectomy, the decision had been made to leave the tumor over her facial nerve and around the stapes alone to avoid facial nerve damage and deafness respectively. At the time, it was not known how aggressive the tumor was and the decision for observation was made.

During the second surgery, meticulous care was taken to remove every part of the tumor around these areas. At the time of this publication the patient is approximately 18 months out from her last surgery and disease free, although time will tell concerning the aggressive nature of her disease. Our original decision to forego radiotherapy on this patient was made due to her young age and the lack of supporting evidence in the literature. We would have chosen to radiate the area if there had been any evidence of malignant transformation, further erosion, or spread of tumor.

## CONCLUSIONS

We plan to continue following the patient with serial MRIs at six-month intervals and physical exams including nasopharyngoscopy. Additional cases must be identified and published to draw statistically relevant clinical characteristics and responses to various medical and surgical therapies. Future publications that identify different presentation trends, treatment regimens, and surveillance protocols may lead to more evidenced-based patient care for this rare condition. The authors suggest that any patient with chronic ear drainage, hearing loss without obvious cause, or chronic otalgia (i.e. earache) be referred to an otolaryngologist for further evaluation.

### Conflict of Interest

The authors declare no conflict of interest.
